# Poly[[μ_2_-1,2-bis­(imidazol-1-ylmeth­yl)benzene](μ_2_-cyclo­hexane-1,4-dicarboxyl­ato)cobalt(II)]

**DOI:** 10.1107/S160053681000646X

**Published:** 2010-02-24

**Authors:** Min Chen, Min Xing

**Affiliations:** a School of Chemistry and Chemical Engineering, Jiangsu University, Zhenjiang 212013, People’s Republic of China

## Abstract

In the the title compound, [Co(C_8_H_10_O_4_)(C_14_H_14_N_4_)]_*n*_, the Co^II^ atom is four-coordinated by two N atoms from two different 1,2-bis­(imidazol-1-ylmeth­yl)benzene ligands and two carboxyl­ate O atoms from two different cyclo­hexane-1,4-dicarboxyl­ate anions in a tetra­hedral coordination geometry. The resulting structure is a two-dimensional polymer with layers in the (100) plane.

## Related literature

For a related structure, see: Li *et al.* (2009 ▶).
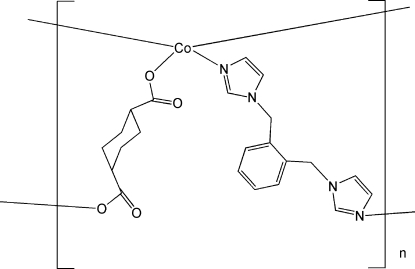


## Experimental

### 

#### Crystal data

[Co(C_8_H_10_O_4_)(C_14_H_14_N_4_)]
*M* *_r_* = 467.38
Monoclinic, 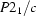

*a* = 9.785 (3) Å
*b* = 12.356 (2) Å
*c* = 17.850 (4) Å
β = 99.559 (2)°
*V* = 2128.2 (9) Å^3^
*Z* = 4
Mo *K*α radiation
μ = 0.84 mm^−1^
*T* = 293 K
0.27 × 0.21 × 0.17 mm

#### Data collection

Oxford Diffraction Gemini R Ultra diffractometer
Absorption correction: multi-scan (*CrysAlis RED*; Oxford Diffraction, 2006 ▶) *T*_min_ = 0.51, *T*_max_ = 0.83
9866 measured reflections
4923 independent reflections
3485 reflections with *I* > 2σ(*I*)
*R*_int_ = 0.019

#### Refinement

*R*[*F* ^2^ > 2σ(*F* ^2^)] = 0.031
*wR*(*F* ^2^) = 0.079
*S* = 0.96
4923 reflections
280 parameters
H-atom parameters constrained
Δρ_max_ = 0.22 e Å^−3^
Δρ_min_ = −0.36 e Å^−3^

### 

Data collection: *CrysAlis CCD* (Oxford Diffraction, 2006 ▶); cell refinement: *CrysAlis RED* (Oxford Diffraction, 2006 ▶); data reduction: *CrysAlis RED*; program(s) used to solve structure: *SHELXS97* (Sheldrick, 2008 ▶); program(s) used to refine structure: *SHELXL97* (Sheldrick, 2008 ▶); molecular graphics: *SHELXTL* (Sheldrick, 2008 ▶); software used to prepare material for publication: *SHELXTL*.

## Supplementary Material

Crystal structure: contains datablocks global, I. DOI: 10.1107/S160053681000646X/bt5196sup1.cif

Structure factors: contains datablocks I. DOI: 10.1107/S160053681000646X/bt5196Isup2.hkl

Additional supplementary materials:  crystallographic information; 3D view; checkCIF report

## Figures and Tables

**Fig. 1.**
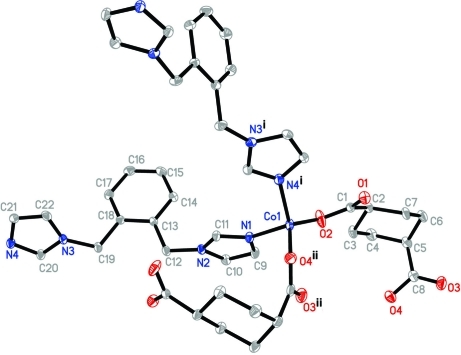
The structure of the title compound, showing the atomic numbering scheme. Displacement ellipsoids are drawn at the 30% probability level. Symmetry codes: (i) x, -0.5-y, z-0.5; (ii) 1-x, y-0.5, 0.5-z.

## supplementary crystallographic information

### Comment

The flexible N-donor ligand,  1,2-bis(imidazol-1-ylmethyl)benzene is a good candidate for the construction of coordination polymers (Li *et al.*, 2009). We report here the synthesis and structure of the title compound, composed of this ligand, cyclohexane-1,4-dicarboxylate and Co atoms.

The Co^II^ atom is four-coordinated by two nitrogen atoms from two different 1,2-bis(imidazol-1-ylmethyl)benzene   ligands and two carboxylate oxygen atoms from two different cyclohexane-1,4-dicarboxylate anions in a tetrahedral coordination geometry. The resulting structure is a two-dimensional polymer with layers in the (1 0 0) plane.

### Experimental

1,4-H_2_chdc (0.5 mmol), 1,2-bix (0.5 mmol) and cobalt chloride hexahydrate (0.5 mmol) were placed in water (12 ml), and triethylamine was added until the pH value of the solution was 5.4. The solution was heated in a 23 ml Teflon-lined stainless-steel autoclave at 430 K for 5 days. The autoclave was cooled to room temperature over several hours. Purple crystals were isolated in about 38% yield.

### Refinement

All H atoms  were positioned geometrically (C—H = 0.93 to 0.98 Å) and refined as riding, with U_iso_(H)=1.2U_eq_(C).

### Crystal data

**Table:**

[Co(C_8_H_10_O_4_)(C_14_H_14_N_4_)]	*F*(000) = 972
*M**_r_* = 467.38	*D*_x_ = 1.459 Mg m^−^^3^
Monoclinic, *P*2_1_/*c*	Mo *K*α radiation, λ = 0.71073 Å
Hall symbol: -P 2ybc	Cell parameters from 4923 reflections
*a* = 9.785 (3) Å	θ = 2.3–29.1°
*b* = 12.356 (2) Å	µ = 0.84 mm^−^^1^
*c* = 17.850 (4) Å	*T* = 293 K
β = 99.559 (2)°	Block, purple
*V* = 2128.2 (9) Å^3^	0.27 × 0.21 × 0.17 mm
*Z* = 4	

### Data collection

**Table:**

Oxford Diffraction Gemini R Ultra diffractometer	4923 independent reflections
Radiation source: fine-focus sealed tube	3485 reflections with *I* > 2σ(*I*)
graphite	*R*_int_ = 0.019
Detector resolution: 10.0 pixels mm^-1^	θ_max_ = 29.1°, θ_min_ = 2.3°
ω scan	*h* = −8→12
Absorption correction: multi-scan (*CrysAlis RED*; Oxford Diffraction, 2006)	*k* = −16→15
*T*_min_ = 0.51, *T*_max_ = 0.83	*l* = −23→22
9866 measured reflections	

### Refinement

**Table:**

Refinement on *F*^2^	Primary atom site location: structure-invariant direct methods
Least-squares matrix: full	Secondary atom site location: difference Fourier map
*R*[*F*^2^ > 2σ(*F*^2^)] = 0.031	Hydrogen site location: inferred from neighbouring sites
*wR*(*F*^2^) = 0.079	H-atom parameters constrained
*S* = 0.96	*w* = 1/[σ^2^(*F*_o_^2^) + (0.0456*P*)^2^] where *P* = (*F*_o_^2^ + 2*F*_c_^2^)/3
4923 reflections	(Δ/σ)_max_ = 0.001
280 parameters	Δρ_max_ = 0.22 e Å^−^^3^
0 restraints	Δρ_min_ = −0.36 e Å^−^^3^

### Special details

**Table:**

Geometry. All esds (except the esd in the dihedral angle between two l.s. planes) are estimated using the full covariance matrix. The cell esds are taken into account individually in the estimation of esds in distances, angles and torsion angles; correlations between esds in cell parameters are only used when they are defined by crystal symmetry. An approximate (isotropic) treatment of cell esds is used for estimating esds involving l.s. planes.
Refinement. Refinement of *F*^2^ against ALL reflections. The weighted *R*-factor wR and goodness of fit *S* are based on *F*^2^, conventional *R*-factors *R* are based on *F*, with *F* set to zero for negative *F*^2^. The threshold expression of *F*^2^ > σ(*F*^2^) is used only for calculating *R*-factors(gt) etc. and is not relevant to the choice of reflections for refinement. *R*-factors based on *F*^2^ are statistically about twice as large as those based on *F*, and *R*- factors based on ALL data will be even larger.

### Fractional atomic coordinates and isotropic or equivalent isotropic displacement parameters (Å^2^)

**Table:**

	*x*	*y*	*z*	*U*_iso_*/*U*_eq_	
C1	0.73125 (18)	0.35656 (12)	0.18015 (9)	0.0353 (4)	
C2	0.82011 (19)	0.44982 (12)	0.21681 (8)	0.0372 (4)	
H2	0.9170	0.4315	0.2151	0.045*	
C3	0.8064 (2)	0.46346 (14)	0.30090 (9)	0.0452 (4)	
H3A	0.7090	0.4687	0.3049	0.054*	
H3B	0.8434	0.3996	0.3287	0.054*	
C4	0.8814 (2)	0.56275 (15)	0.33757 (10)	0.0515 (5)	
H4A	0.9805	0.5529	0.3407	0.062*	
H4B	0.8621	0.5705	0.3889	0.062*	
C5	0.8367 (2)	0.66551 (14)	0.29255 (10)	0.0476 (5)	
H5	0.8949	0.7248	0.3162	0.057*	
C6	0.8670 (2)	0.65185 (14)	0.21139 (10)	0.0513 (5)	
H6A	0.8402	0.7171	0.1824	0.062*	
H6B	0.9657	0.6411	0.2130	0.062*	
C7	0.7878 (2)	0.55525 (13)	0.17234 (9)	0.0450 (5)	
H7A	0.8115	0.5467	0.1220	0.054*	
H7B	0.6892	0.5696	0.1665	0.054*	
C8	0.6860 (2)	0.69815 (14)	0.29141 (10)	0.0460 (5)	
C9	0.64364 (19)	0.15946 (12)	0.36882 (9)	0.0393 (4)	
H9	0.6565	0.2340	0.3690	0.047*	
C10	0.65119 (17)	0.09703 (13)	0.43108 (8)	0.0368 (4)	
H10	0.6699	0.1200	0.4814	0.044*	
C11	0.60365 (17)	−0.00366 (12)	0.32978 (8)	0.0340 (4)	
H11	0.5836	−0.0639	0.2986	0.041*	
C12	0.62805 (17)	−0.10377 (13)	0.45236 (9)	0.0375 (4)	
H12A	0.5627	−0.1555	0.4258	0.045*	
H12B	0.5966	−0.0851	0.4995	0.045*	
C13	0.76758 (16)	−0.15711 (12)	0.47099 (8)	0.0294 (3)	
C14	0.88512 (18)	−0.11324 (14)	0.44907 (9)	0.0379 (4)	
H14	0.8783	−0.0491	0.4214	0.046*	
C15	1.01216 (19)	−0.16348 (15)	0.46771 (11)	0.0478 (5)	
H15	1.0900	−0.1343	0.4517	0.057*	
C16	1.02296 (19)	−0.25688 (16)	0.51008 (11)	0.0488 (5)	
H16	1.1089	−0.2898	0.5239	0.059*	
C17	0.90668 (18)	−0.30224 (14)	0.53235 (9)	0.0409 (4)	
H17	0.9148	−0.3657	0.5608	0.049*	
C18	0.77854 (16)	−0.25356 (12)	0.51251 (8)	0.0304 (3)	
C19	0.64901 (18)	−0.30062 (13)	0.53515 (10)	0.0426 (4)	
H19A	0.6259	−0.2593	0.5775	0.051*	
H19B	0.5732	−0.2925	0.4930	0.051*	
C20	0.64394 (19)	−0.45501 (12)	0.62419 (8)	0.0382 (4)	
H20	0.6285	−0.4133	0.6653	0.046*	
C21	0.67567 (18)	−0.58996 (13)	0.55355 (9)	0.0407 (4)	
H21	0.6869	−0.6603	0.5371	0.049*	
C22	0.67997 (19)	−0.50032 (14)	0.51112 (9)	0.0433 (4)	
H22	0.6934	−0.4972	0.4608	0.052*	
O1	0.69190 (13)	0.35191 (9)	0.11146 (6)	0.0465 (3)	
O2	0.69910 (18)	0.28649 (10)	0.22607 (7)	0.0728 (5)	
O3	0.63128 (16)	0.76656 (10)	0.24541 (8)	0.0623 (4)	
O4	0.61815 (14)	0.65379 (10)	0.33933 (7)	0.0507 (3)	
Co1	0.58188 (3)	0.163209 (16)	0.199462 (11)	0.03716 (9)	
N1	0.61394 (14)	0.09605 (10)	0.30478 (7)	0.0355 (3)	
N2	0.62601 (13)	−0.00679 (10)	0.40605 (6)	0.0320 (3)	
N3	0.66068 (14)	−0.41468 (10)	0.55676 (7)	0.0363 (3)	
N4	0.65211 (15)	−0.56183 (10)	0.62477 (7)	0.0376 (3)	

### Atomic displacement parameters (Å^2^)

**Table:**

	*U*^11^	*U*^22^	*U*^33^	*U*^12^	*U*^13^	*U*^23^
C1	0.0446 (10)	0.0288 (8)	0.0349 (8)	0.0005 (7)	0.0134 (7)	−0.0012 (7)
C2	0.0450 (10)	0.0350 (9)	0.0322 (8)	−0.0061 (8)	0.0081 (7)	−0.0004 (7)
C3	0.0651 (13)	0.0405 (9)	0.0284 (8)	−0.0088 (9)	0.0029 (8)	0.0044 (7)
C4	0.0632 (13)	0.0539 (11)	0.0357 (9)	−0.0103 (10)	0.0036 (9)	−0.0056 (8)
C5	0.0629 (13)	0.0392 (9)	0.0427 (10)	−0.0196 (9)	0.0144 (9)	−0.0098 (8)
C6	0.0732 (15)	0.0381 (10)	0.0474 (10)	−0.0159 (9)	0.0239 (10)	−0.0020 (8)
C7	0.0710 (14)	0.0358 (9)	0.0299 (8)	−0.0082 (9)	0.0136 (8)	0.0033 (7)
C8	0.0704 (14)	0.0320 (8)	0.0376 (9)	−0.0129 (9)	0.0144 (9)	−0.0138 (8)
C9	0.0589 (12)	0.0271 (8)	0.0317 (8)	−0.0047 (8)	0.0065 (8)	−0.0034 (7)
C10	0.0442 (10)	0.0390 (9)	0.0261 (7)	−0.0042 (8)	0.0028 (7)	−0.0037 (7)
C11	0.0484 (11)	0.0268 (7)	0.0267 (7)	−0.0009 (7)	0.0057 (7)	−0.0001 (6)
C12	0.0350 (10)	0.0401 (9)	0.0376 (8)	−0.0006 (7)	0.0064 (7)	0.0152 (7)
C13	0.0305 (9)	0.0332 (8)	0.0241 (7)	−0.0033 (7)	0.0036 (6)	0.0005 (6)
C14	0.0377 (10)	0.0389 (9)	0.0377 (8)	−0.0064 (8)	0.0077 (7)	0.0040 (7)
C15	0.0334 (11)	0.0568 (11)	0.0551 (11)	−0.0066 (9)	0.0132 (9)	−0.0003 (9)
C16	0.0302 (11)	0.0564 (11)	0.0590 (11)	0.0073 (9)	0.0050 (8)	−0.0021 (9)
C17	0.0405 (11)	0.0386 (9)	0.0421 (9)	0.0045 (8)	0.0023 (8)	0.0042 (8)
C18	0.0327 (9)	0.0321 (8)	0.0264 (7)	−0.0001 (7)	0.0052 (6)	0.0009 (6)
C19	0.0405 (11)	0.0357 (8)	0.0533 (10)	0.0041 (8)	0.0127 (8)	0.0184 (8)
C20	0.0537 (11)	0.0320 (8)	0.0305 (8)	0.0004 (8)	0.0114 (7)	0.0034 (7)
C21	0.0508 (11)	0.0338 (8)	0.0413 (9)	−0.0012 (8)	0.0187 (8)	−0.0043 (7)
C22	0.0542 (12)	0.0466 (10)	0.0337 (8)	−0.0053 (9)	0.0201 (8)	−0.0009 (8)
O1	0.0559 (8)	0.0480 (7)	0.0332 (6)	−0.0070 (6)	0.0002 (6)	−0.0032 (5)
O2	0.1385 (15)	0.0422 (7)	0.0405 (7)	−0.0395 (9)	0.0230 (8)	−0.0041 (6)
O3	0.0897 (11)	0.0376 (7)	0.0610 (8)	0.0019 (7)	0.0166 (8)	0.0034 (6)
O4	0.0642 (9)	0.0535 (8)	0.0360 (6)	−0.0124 (6)	0.0131 (6)	−0.0060 (5)
Co1	0.06634 (19)	0.02305 (11)	0.02269 (11)	−0.00140 (10)	0.00917 (10)	−0.00115 (8)
N1	0.0525 (9)	0.0282 (7)	0.0257 (6)	−0.0011 (6)	0.0065 (6)	0.0003 (5)
N2	0.0348 (8)	0.0334 (7)	0.0274 (6)	−0.0003 (6)	0.0037 (5)	0.0080 (5)
N3	0.0443 (9)	0.0333 (7)	0.0331 (6)	−0.0007 (6)	0.0115 (6)	0.0086 (6)
N4	0.0510 (9)	0.0309 (7)	0.0317 (7)	0.0005 (6)	0.0089 (6)	0.0037 (6)

### Geometric parameters (Å, °)

**Table:**

C1—O1	1.2240 (19)	C12—C13	1.503 (2)
C1—O2	1.2673 (19)	C12—H12A	0.9700
C1—C2	1.524 (2)	C12—H12B	0.9700
C2—C7	1.531 (2)	C13—C14	1.385 (2)
C2—C3	1.538 (2)	C13—C18	1.398 (2)
C2—H2	0.9800	C14—C15	1.380 (2)
C3—C4	1.521 (2)	C14—H14	0.9300
C3—H3A	0.9700	C15—C16	1.374 (3)
C3—H3B	0.9700	C15—H15	0.9300
C4—C5	1.527 (3)	C16—C17	1.385 (3)
C4—H4A	0.9700	C16—H16	0.9300
C4—H4B	0.9700	C17—C18	1.383 (2)
C5—C8	1.526 (3)	C17—H17	0.9300
C5—C6	1.536 (3)	C18—C19	1.509 (2)
C5—H5	0.9800	C19—N3	1.461 (2)
C6—C7	1.527 (2)	C19—H19A	0.9700
C6—H6A	0.9700	C19—H19B	0.9700
C6—H6B	0.9700	C20—N4	1.3222 (19)
C7—H7A	0.9700	C20—N3	1.3377 (19)
C7—H7B	0.9700	C20—H20	0.9300
C8—O3	1.237 (2)	C21—C22	1.346 (2)
C8—O4	1.289 (2)	C21—N4	1.374 (2)
C9—C10	1.345 (2)	C21—H21	0.9300
C9—N1	1.376 (2)	C22—N3	1.367 (2)
C9—H9	0.9300	C22—H22	0.9300
C10—N2	1.367 (2)	O2—Co1	1.9185 (13)
C10—H10	0.9300	O4—Co1^i^	1.9682 (15)
C11—N1	1.3199 (19)	Co1—O4^ii^	1.9682 (15)
C11—N2	1.3432 (18)	Co1—N4^iii^	2.0306 (13)
C11—H11	0.9300	Co1—N1	2.0311 (12)
C12—N2	1.4541 (19)	N4—Co1^iv^	2.0306 (13)
			
O1—C1—O2	123.04 (15)	C13—C12—H12B	108.7
O1—C1—C2	121.85 (14)	H12A—C12—H12B	107.6
O2—C1—C2	115.09 (14)	C14—C13—C18	119.29 (15)
C1—C2—C7	111.52 (13)	C14—C13—C12	122.16 (14)
C1—C2—C3	111.46 (13)	C18—C13—C12	118.55 (14)
C7—C2—C3	111.64 (13)	C15—C14—C13	120.84 (16)
C1—C2—H2	107.3	C15—C14—H14	119.6
C7—C2—H2	107.3	C13—C14—H14	119.6
C3—C2—H2	107.3	C16—C15—C14	119.63 (17)
C4—C3—C2	113.23 (14)	C16—C15—H15	120.2
C4—C3—H3A	108.9	C14—C15—H15	120.2
C2—C3—H3A	108.9	C15—C16—C17	120.41 (17)
C4—C3—H3B	108.9	C15—C16—H16	119.8
C2—C3—H3B	108.9	C17—C16—H16	119.8
H3A—C3—H3B	107.7	C18—C17—C16	120.26 (16)
C3—C4—C5	111.62 (14)	C18—C17—H17	119.9
C3—C4—H4A	109.3	C16—C17—H17	119.9
C5—C4—H4A	109.3	C17—C18—C13	119.54 (15)
C3—C4—H4B	109.3	C17—C18—C19	122.00 (14)
C5—C4—H4B	109.3	C13—C18—C19	118.46 (14)
H4A—C4—H4B	108.0	N3—C19—C18	114.22 (14)
C8—C5—C4	114.75 (16)	N3—C19—H19A	108.7
C8—C5—C6	110.77 (16)	C18—C19—H19A	108.7
C4—C5—C6	108.75 (15)	N3—C19—H19B	108.7
C8—C5—H5	107.4	C18—C19—H19B	108.7
C4—C5—H5	107.4	H19A—C19—H19B	107.6
C6—C5—H5	107.4	N4—C20—N3	111.26 (14)
C7—C6—C5	110.70 (14)	N4—C20—H20	124.4
C7—C6—H6A	109.5	N3—C20—H20	124.4
C5—C6—H6A	109.5	C22—C21—N4	109.76 (14)
C7—C6—H6B	109.5	C22—C21—H21	125.1
C5—C6—H6B	109.5	N4—C21—H21	125.1
H6A—C6—H6B	108.1	C21—C22—N3	106.33 (14)
C6—C7—C2	112.53 (14)	C21—C22—H22	126.8
C6—C7—H7A	109.1	N3—C22—H22	126.8
C2—C7—H7A	109.1	C1—O2—Co1	125.78 (11)
C6—C7—H7B	109.1	C8—O4—Co1^i^	109.44 (12)
C2—C7—H7B	109.1	O2—Co1—O4^ii^	130.75 (6)
H7A—C7—H7B	107.8	O2—Co1—N4^iii^	113.50 (6)
O3—C8—O4	121.2 (2)	O4^ii^—Co1—N4^iii^	99.08 (6)
O3—C8—C5	120.01 (17)	O2—Co1—N1	95.80 (5)
O4—C8—C5	118.80 (17)	O4^ii^—Co1—N1	107.05 (5)
C10—C9—N1	109.56 (14)	N4^iii^—Co1—N1	109.79 (5)
C10—C9—H9	125.2	C11—N1—C9	105.55 (12)
N1—C9—H9	125.2	C11—N1—Co1	133.27 (10)
C9—C10—N2	106.62 (13)	C9—N1—Co1	121.00 (10)
C9—C10—H10	126.7	C11—N2—C10	107.12 (12)
N2—C10—H10	126.7	C11—N2—C12	125.77 (13)
N1—C11—N2	111.14 (13)	C10—N2—C12	127.07 (12)
N1—C11—H11	124.4	C20—N3—C22	107.30 (13)
N2—C11—H11	124.4	C20—N3—C19	125.54 (14)
N2—C12—C13	114.42 (13)	C22—N3—C19	127.00 (13)
N2—C12—H12A	108.7	C20—N4—C21	105.34 (13)
C13—C12—H12A	108.7	C20—N4—Co1^iv^	126.46 (11)
N2—C12—H12B	108.7	C21—N4—Co1^iv^	125.35 (11)

Symmetry codes: (i) −*x*+1, *y*+1/2, −*z*+1/2; (ii) −*x*+1, *y*−1/2, −*z*+1/2; (iii) *x*, −*y*−1/2, *z*−1/2; (iv) *x*, −*y*−1/2, *z*+1/2.
